# A Retrospective Observational Study of the Impact of HIV Status on the Outcome of Paediatric Intensive Care Unit Admissions at a Tertiary Hospital in South Africa (2015–2019)

**DOI:** 10.3390/pediatric15040061

**Published:** 2023-11-08

**Authors:** Kim Whitehead, Daynia E. Ballot

**Affiliations:** Department of Paediatrics and Child Health, Faculty of Health Sciences, University of the Witwatersrand, Johannesburg 2050, South Africa; daynia.ballot@wits.ac.za

**Keywords:** human immunodeficiency virus, paediatric intensive care unit, outcomes

## Abstract

HIV-infected and HIV-exposed but uninfected (HEU) children have unique health risks. Our study looked at how HIV exposure and infection impact presentation and outcomes in PICU in an era of improved ART. A retrospective analysis of children admitted to PICU was performed. The sample was divided into HIV negative, HEU and HIV infected, and presentation and outcomes were compared with a significance level set at α = 0.05. Our study showed that 16% (109/678) of children admitted to PICU were HEU and 5.2% (35/678) were HIV infected. HIV-infected children were admitted at a younger age (median two months) with an increased incidence of lower respiratory infections than HIV-negative children (*p* < 0.001); they also required longer ventilation and admission (*p* < 0.001). HIV-infected children had a higher mortality (40%) (*p* = 0.02) than HIV-negative (22.7%) children; this difference was not significant when comparing only children with a non-surgical diagnosis (*p* = 0.273). HEU children had no significant difference in duration of ICU stay (*p* = 0.163), ventilation (*p* = 0.443) or mortality (*p* = 0.292) compared to HIV-negative children. In conclusion, HIV-infected children presented with more severe disease requiring longer ventilation and admission. HEU had similar outcomes to HIV-negative children.

## 1. Introduction

Human immunodeficiency virus (HIV) remains an important healthcare problem. Despite the introduction of expanded Prevention of Mother-to-Child Transmission (PMTCT) programmes and universal access to Antiretroviral Therapy (ART), HIV still accounts for 1.2–8.7% of under-five deaths in South Africa [1]. South Africa has reduced its mother-to-child transmission from 16% in 2010 to 3.3% in 2019 resulting in a growing population of children who are HIV-exposed but uninfected (HEU) [2,3]. In 2019, there were an estimated 290,000 children living with HIV, 3700 HIV-related deaths in children and 3.9 million HEU children in South Africa [2].

Currently, in South Africa, only 51% of HIV-infected children are on ART [2]. HIV-infected children not yet on treatment often present to a healthcare facility with an acute severe illness requiring Paediatric Intensive Care Unit (PICU) admission as their first presentation [4,5]. At the start of the HIV epidemic, admission of HIV-infected children into PICU was considered futile in view of a mortality as high as 84–100%, ventilation showing no benefit and there being little hope of cure [6,7,8]. As the epidemic evolved, ART became more readily available, and treatment and ventilation strategies for opportunistic infections improved. This led to improved outcomes with several studies showing no difference in mortality between HIV-infected and HIV-negative children [4,5,9,10,11]. In view of this, HIV is no longer a reason to deny a child admission to PICU [5,12].

HIV-infected children are most often admitted to PICU with acute respiratory tract infections but may also present with multi-organ involvement. Opportunistic infections such as pneumocystis pneumonia (PCP) and cytomegalovirus (CMV) increase mortality to up to 50% despite the availability of antimicrobials for these diseases [4,13,14,15]. The growing population of HEU children is also important as HIV exposure increases the risk for severe pneumonia and opportunistic infections with a proportion of these children requiring PICU admission [16]. Although HIV exposure has not been shown to increase mortality in PICU, these patients may have a significantly longer stay in PICU [10,11]. This is likely to place an additional burden on limited PICU resources.

PICU is a limited resource in South Africa, with only 19.6% of public ICU beds allocated to neonatal and paediatric patients [17]. Ethical dilemmas arise regarding which patients to accept to PICU in view of the limited beds and occupancy levels of 97% [12]. There are currently no national guidelines on the allocation of PICU beds; therefore, hospitals draw on international guidelines or set up their own admission guidelines [12]. There is a need for low- and middle-income countries (LMICs) to develop their own critical care guidelines based on available resources and disease spectrum while being guided by the local context [18].

Statistics on the mortality rate in HIV-infected children admitted to PICU are still conflicting, ranging from 11 to 31.3% depending on the centre [9,10,11]. Further studies are required to ascertain how HIV infection and exposure impact on presentation and outcomes in PICU in the era of improved access to ART and PMTCT. This will allow for more resources for PICU and HIV programmes and assist with drawing up PICU admission guidelines. The aim of this study was therefore to compare the characteristics and outcomes of HIV-negative, HEU and HIV-infected children admitted to PICU.

## 2. Materials and Methods

### 2.1. Study Design

This was a quantitative, observational study with data collected retrospectively from 2015 to 2019. The study was a secondary analysis of an existing database. The database was created by capturing data on discharge from PICU for the purpose of quality improvement and managed by the Research Electronic Data Capture (REDCap) system hosted by the University of the Witwatersrand [19]. The data were collected on discharge of patients with several checkpoints.

### 2.2. Study Setting

The study was based at the PICU at Charlotte Maxeke Johannesburg Academic Hospital (CMJAH) in Johannesburg, South Africa. CMJAH is a tertiary/quaternary hospital that provides healthcare to the Gauteng population as well as some of the neighbouring provinces and countries where tertiary services are not available. At the time of the study, CMJAH had 14 shared neonatal and PICU beds; these beds are in high demand by both medical and surgical specialities. Usually, only children requiring invasive ventilation are admitted to this centre’s PICU.

### 2.3. Participants

All children between the age of 29 days and 12 years 11 months admitted to the PICU at CMJAH between January 2015 and December 2019 were eligible for inclusion in this study. Children with missing records or for whom the HIV results were unknown were excluded. If children were readmitted, each admission was counted separately in the study.

### 2.4. Study Procedure

We retrieved de-identified data for the study period from the CMJAH PICU database. De-identified data were imported into an Excel spreadsheet, and the inclusion and exclusion criteria were applied.

We captured demographic data, namely gender and admission age in months. We classified the HIV status as follows [3,20]:

(1) HIV negative: The mother was negative, or the child had a negative HIV ELISA;
(2) HEU: The mother was HIV positive, or the child less than 18 months had a positive ELISA AND the child had a negative HIV PCR;
(3) HIV infected: A child less than 18 months with a positive HIV PCR or a child more than 18 months with a positive ELISA. HIV infection was confirmed with a second ELISA, PCR or HIV viral load.

#### HIV-Exposed Children Include Both HEU and HIV-Infected Children

We captured clinical data consisting of weight, height, admission diagnosis, ventilation and inotropic support, opportunistic and other infections. Weight z-scores were calculated using weight in kilograms and WHO growth charts [21]. Height was not analysed as it was often not recorded. The admission diagnosis was the reason for requiring ICU and was classified into 4 groups:

(1) Surgical: Any patient admitted with an underlying surgical condition;
(2) Accidents (including trauma), drownings and poisonings (A, D + P);
(3) Lower respiratory tract (LRT) conditions: An illness affecting the respiratory tract below the level of the larynx including pneumonia, bronchiolitis, asthma and pleural effusions;
(4) Other medical conditions: Medical conditions other than lower respiratory tract conditions (e.g., gastrointestinal and cardiovascular conditions).

Patients were classified as being post-operative if they were admitted to the PICU after a surgical procedure. The ventilatory mode was defined as the maximum level of ventilatory support required: continuous positive airway pressure (CPAP), intermittent positive pressure ventilation (IPPV) or high-frequency ventilation (HFOV).

Opportunistic and other infections were diagnosed as follows. Pneumocystis pneumonia (PCP) was a clinical diagnosis supported by laboratory markers such as lactate dehydrogenase (LDH), beta-D-glucan and *Pneumocystis jirovecii* polymerase chain reaction (PCR). Cytomegalovirus (CMV) was suspected clinically in HIV-exposed or infected children admitted with severe pneumonia and confirmed with a CMV viral load of more than 10,000. Tuberculosis was diagnosed either on clinical and X-ray findings or with a GeneXpert and TB microscopy and culture. Bacterial and fungal infections were defined by positive blood cultures. Viral infections (excluding HIV) included influenza, respiratory syncytial virus (RSV), adenovirus and other respiratory viruses as well as CMV and were confirmed by viral PCR on respiratory swabs and CMV viral loads.

The outcome measures we looked at were duration of ventilation, length of PICU admission and death in PICU.

The study population was then divided into three groups, namely HIV infected, HEU and HIV negative. A comparison of the demographics, clinical data and outcomes was performed between these three groups. Further analysis was then conducted by excluding children admitted with a surgical condition or due to accidents, drownings and poisonings, as the majority of these groups did not have HIV, and this could bias the sample.

### 2.5. Statistical Methods

Data analysis was performed using SPSS software (Version 27). Only valid cases were analysed for each variable (i.e., missing information was excluded). For continuous variables, descriptive statistics were computed based on the variable’s distribution characteristics (i.e., medians and interquartile ranges or means and standard deviations). Frequencies were used to describe categorical variables.

The comparison between the HIV-negative, HEU and HIV-infected groups was conducted using the Chi-squared test to compare categorical variables. If there was a significant difference, a second analysis between HIV-infected and HIV-negative groups, HEU and HIV-infected groups and HEU and HIV-negative groups was conducted separately to see which groups accounted for the difference. The non-parametric Kruskal–Wallis test was used to compare continuous variables between groups using the Bonferroni correction. For all statistical tests, we used a significance level of α = 0.05.

#### Ethical Consideration

The Human Research Ethics Committee of the University of the Witwatersrand (clearance certificate M200436) as well as the clinical manager at CMJAH approved the study. Due to the retrospective nature of the study, the need for informed consent was waived by the Human Research Ethics Committee of the University of the Witwatersrand.

## 3. Results

### 3.1. Descriptive Statistics

**Figure 1:**
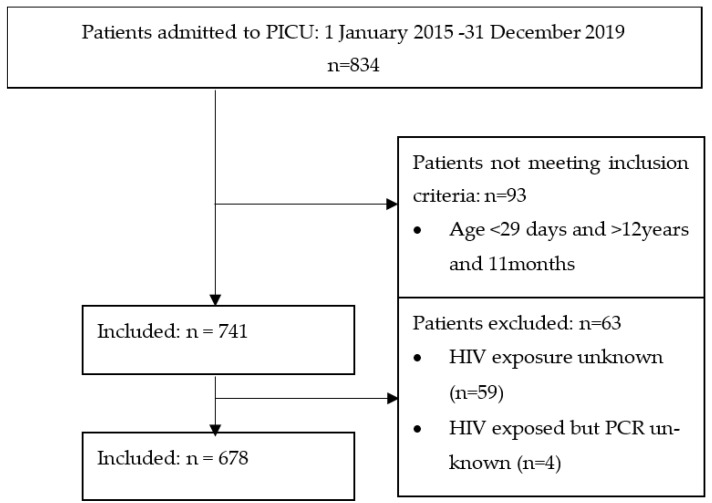
Flow diagram of the selection of participants.

A total of 834 children were admitted to the PICU at CMJAH, from 1 January 2015 to 31 December 2019. Our final sample size was 678 (see Figure 1).

The HIV status of the participants in the study was as follows: 534/678 (78.8%) were HIV negative, and 144/678 (21.2%) were HIV exposed. In the HIV-exposed group, 35/144 (24.3%) were HIV infected and 109/144 (75.7%) were HIV exposed but uninfected (HEU). In total, 5.2% (35/678) of the admissions were HIV infected.

The median age of the children admitted to the PICU was 8 months with an interquartile range (IQR) of 42 months (the first quartile (Q1) was 2 months, and the third quartile (Q3) was 44 months). Females made up 46.8% (317/678) of the sample; males made up 53.1% (360/678), and the remaining 0.1% (1/678) were intersex. The median weight z-score of children admitted to PICU was −1.73 (IQR 3.44 (Q1 −0.23; Q3 −3.67)).

Patients admitted to the PICU had a wide variety of diagnoses on admission with the majority having a primary surgical diagnosis 298/678 (44%), followed by lower respiratory tract conditions 172/678 (25.4%). The remainder of patients were admitted with other medical conditions (121/678 (17.8%)), followed by accidents, poisonings and drownings (87/678 (12.8%)). Forty eight percent (317/668) of patients were admitted to PICU post-operatively.

Most patients, 619/673 (92%) were intubated and ventilated. The majority were treated with IPPV 532/654 (81.3%), while 43/654 (6.6%) required HFOV and 19/654 (2.9%) required CPAP. Inotropic support was given to 129/671 (19.2%) patients.

Bacterial infections were diagnosed in 115 patients (17.6%), viral infections in 51 patients (7.8%) and fungal infections in 14 patients (2.1%). The most common organisms cultured were Coagulase Negative Staphylococcus (CNS) (these were likely to have been contaminants) followed by *Staphylococcus aureus* and Gram-negative organisms (*Acinetobacter* species and *Klebsiella* species). The most common viral infection was respiratory syncytial virus (21/657, 3.2%), and multiple viral organisms were found in 3/657 (0.5%) patients.

Patients were ventilated and admitted to PICU for a median of three days (IQR of eight). The mortality rate in PICU was 24.8% (167/673) during our study period.

### 3.2. Comparison between HIV-Negative, HEU and HIV-Infected Patients

HIV status had an association with age on admission (*p* < 0.001), weight z-score (*p* < 0.001) and admission diagnosis (*p* < 0.001)) when doing an overall comparison between the three groups. HIV status was also associated with the occurrence of TB (*p* < 0.001), viral infections (*p* < 0.001) and PCP/CMV (*p* < 0.001). The median duration of ventilation (*p* < 0.001), median length of ICU stay (*p* < 0.001) and mortality (*p* = 0.025) were significantly different between the HIV status groups. However, no significant differences were found between the three groups for gender (*p* = 0.988), incidence of bacterial (*p* = 0.160) or fungal (*p* = 0.645) infections and need for ventilation (*p* = 0.179) or inotropic support (*p* = 0.689). Table 1, Table 2 and Table 3 compare HIV-negative, HIV-infected and HEU groups for variables where there was a significant overall *p*-value between the three groups.

HIV-infected children tended to be younger on admission than HIV-negative but not HEU children. HIV-infected children were more frequently admitted with an LRT condition and more frequently diagnosed with opportunistic (PCP/CMV/TB) and viral infections than HIV-negative and HEU children. HIV-infected children had a worse outcome than HIV-negative and HEU children requiring HFOV more often and being ventilated and admitted to PICU for longer. The mortality in HIV-infected children (40%) was higher than the mortality in HIV-negative children (22.7%) (*p* = 0.02). Although the mortality rate in HIV-infected children was higher than in HEU (30.3%), this difference was not statistically significant (*p* = 0.286).

HEU children were younger and had a lower weight for age z-score than HIV-negative children. HEU children were more frequently admitted with LRT conditions and more frequently diagnosed with PCP and CMV; however, they did not have a higher incidence of TB compared to HIV-negative children. HEU children had a similar outcome to HIV-negative children, requiring a similar duration of ventilation and ICU admission. The difference in mortality between HEU and HIV-negative children was not statistically significant (*p* = 0.091).

### 3.3. Comparison by HIV Status for Children Admitted with a Medical Diagnosis

The subsequent comparison of patients admitted with a medical condition (lower respiratory tract or other) showed that 11.3% (33/293) of children were HIV positive. HIV-exposed children made up 34.5% (101/293) of total medical admissions, of which 67.3% (68/101) were HEU and 32.7% (33/101) were HIV infected. The comparison of demographics and outcomes are shown in Table 4.

When looking at the differences between the individual groups, we found that HIV-infected and HEU children were significantly younger on admission compared to HIV-negative children (*p* = 0.013 and *p* = 0.02). PCP and CMV were more frequently diagnosed in the HIV-infected (*p* < 0.001) and HEU groups (*p* < 0.001) than the HIV-negative group, while TB was only more frequent in HIV-positive children (*p* < 0.001). HIV-infected children required HFOV significantly more often than HIV-negative children (*p* = 0.04). They also required longer ventilation (*p* = 0.001 and *p* = 0.002) and ICU stay (*p* = 0.001 and *p* = 0.013) than both HIV-negative and HEU children. Although HIV-infected and HEU children had a higher mortality, this difference was not significant (*p* = 0.387).

## 4. Discussion

This study analysed the extent to which outcomes in PICU differ according to HIV status. Despite an aggressive PMTCT programme and widely available ART, our study showed that HIV-infected children made up 5.2% of the total admissions and 11.3% of medical admissions in the PICU. Previous South African studies showed similar proportions, with 10–12% of medical PICU admissions being HIV-infected children [10,11].

Since the introduction of expanded PMTCT programmes in South Africa, fewer HIV-exposed children are sero-converting and becoming HIV infected. This is evidenced in our study as only 32.7% of HIV-exposed children admitted with a medical diagnosis had a positive PCR (i.e., confirmed to be HIV infected). Between 2003 and 2009, this figure was much higher with more than 80% of HIV-exposed children admitted to the PICU being confirmed as being HIV infected [7,14]. With continually improved PMTCT programmes, PICU should hopefully see a further decrease in the number of HIV-infected children requiring admission. Every HIV-infected case should be used as an opportunity to try and identify gaps in the PMTCT programme.

HEU and HIV-infected children required admission to PICU at a younger age (two to three months) than HIV-negative children. This was similar to local and international studies which showed that most HIV-infected children were younger than six months when admitted to PICU [4,11,14,22]. The younger age of admission is postulated to be due to the disease spectrum and severity in these groups. HIV-infected children often present to a healthcare facility for the first time at this age with acute severe disease which may require PICU care [5]. The younger age of admission shows that this age group of children are an important group to target for the prevention of infectious diseases.

Furthermore, our study showed that HEU and HIV-infected children were more frequently admitted with LRT conditions than HIV-negative children. This is similar to other studies, which have shown that up to 90% of HIV-infected children are admitted to PICU with an LRT infection [9,22,23]. The increased risk for LRT conditions in HIV-exposed children is postulated to be related to the effect of HIV in utero on the development of the immune system, decreased maternal antibodies, increased exposure to household infections and even bone marrow suppression due to ART [16,24].

Despite the routine use of trimethoprim-sulfamethoxazole prophylaxis in HEU and HIV-infected infants, pneumocystis pneumonia remained an important cause of disease in the HEU and HIV-infected children in our study. Pneumocystis pneumonia or pneumocystis and CMV co-infection occurred in 65.7% of HIV-infected children and 6.4% of HEU children in our study. Although treating HIV-exposed patients for PCP and CMV has been shown to improve mortality, PCP still has a mortality of up to 44% despite the use of high dose trimethoprim-sulfamethoxazole, steroids and PICU admission [13,14,15]. Co-infection with CMV further increases mortality despite the use of ganciclovir [14]. The high incidence of pneumocystis in our sample was concerning and reflects poor implementation of trimethoprim-sulfamethoxazole prophylaxis. The high incidence of pneumocystis pneumonia and CMV most likely contributed significantly to the mortality in our patients.

HIV-infected children tended to be admitted with more severe disease, as reflected by them more frequently requiring HFOV. In our PICU, HFOV is only used in patients in whom adequate oxygenation and ventilation is not achieved with IPPV; the use of HFOV thus reflects disease severity. Despite having a more severe disease, HIV-infected children did not have an increased need for inotropes, which is likely due to the primary respiratory pathology in the HIV-infected children.

HIV-infected children also required longer admission and ventilation than HEU and HIV-negative children. Similar findings were noted in previous studies at our and other South African sites [10,11,23]. The longer duration of ventilation and PICU stay are important as they impact on already constrained resources and limited PICU beds. The longer PICU stay may also affect long-term outcomes such as mortality, morbidity from chronic lung disease and neurodevelopment [4,22]. The increased length of ventilation and PICU stay could be related to the severity of disease at presentation as well as the underlying immunocompromised state which may put these children at increased risk for nosocomial infections. Further studies are needed on the outcomes of HIV-infected children once discharged form PICU with longer follow-ups.

While HIV-infected children had a higher mortality than HEU and HIV-negative children, this difference was not significant in children admitted with a medical condition. The higher mortality in HIV-infected children overall (including surgical and trauma conditions) can be explained by most HIV-infected children being admitted with medical conditions. Medical conditions tend to have a worse outcome in patients admitted to PICU for post-operative care [25]. The finding that HIV infection does not significantly affect mortality in PICU is supported by other South African studies and is one of the main reasons why HIV infection is no longer an exclusion diagnosis for PICU admission [10,11,14].

Our HIV-infected children had a mortality of 40% during the study period. This is higher than several studies performed in Cape Town and Pretoria. A study from 2015 to 2017 by Ballot et al. found that the higher mortality at our unit was possibly due to the unit admitting more severely ill children as the predicted mortality using the Paediatric Index of Mortality 3(PIM3) score was similar to the actual mortality [25]. Reasons for this higher mortality can also be attributed to almost all the children in our unit being intubated and ventilated (92%), whereas other centres may admit more children to PICU who do not require invasive ventilation [10]. The availability of high flow nasal prong oxygen in our general paediatric wards for LRT conditions has likely decreased the need for invasive ventilation in PICU and led to only the most severely ill children with LRT conditions being admitted to PICU. The high incidence of pneumocystis pneumonia and CMV in our centre also contributes to the higher mortality.

HEU children made up a significant percentage of PICU admissions. The number might in fact be higher as HIV exposure in surgical patients is often not well documented. This number is likely to rise with improved PMTCT. HEU children were admitted at a younger age and had a lower weight-for-age z-score than HIV-negative children. This shows that HEU children are at risk of malnutrition and severe disease at a young age, and special attention should be given to these children when monitoring their growth and nutrition.

Although HEU children had similar outcomes to their HIV-negative counterparts, they seemed to have a higher burden of LRT conditions as well as opportunistic infections such as pneumocystis pneumonia and CMV. Numerous postulates have been made for this including immune dysregulation in HEU children [14]. The higher incidence of pneumocystis in these children shows that there is an opportunity to improve the implementation of trimethoprim-sulfamethoxazole prophylaxis in HEU children. Kitchin et al. found that HEU children in their unit were often not on prophylaxis as the mothers were not aware of HIV exposure [14]. Further studies are needed to establish why HEU children are presenting to PICU at a younger age and on interventions to decrease the risk of acute severe disease in this population, which would decrease the pressure on PICU beds.

The major limitation of our study was that it was a retrospective study of an existing database and not all data were complete. In classifying disease, there was no specific definition used for PCP; rather it was a diagnosis made by the treating team based on clinical grounds supported by investigations. Another major limitation was that we did not have information on the timing of HIV diagnosis, maternal HIV history and PMTCT received by the mother and child which could help identify gaps in the PMTCT programme. The study only used an analysis of differences between groups and no multivariable statistical models were used. Lastly, we did not obtain data on the long-term outcomes of patients.

## 5. Conclusions

In conclusion, our study showed that HIV-infected and HEU children presented to ICU at a younger age and were at increased risk of lower respiratory tract conditions than HIV-negative children. The high incidence of PCP suggests the need to improve the implementation of PCP prophylaxis in HEU and HIV-infected children. HIV-infected children tend to have more severe disease requiring longer ventilation and PICU stay. They also have an increased mortality, but this difference was not significant in children admitted with medical conditions only. This shows that HIV infection on its own should no longer exclude a child from PICU admission. HEU children had similar outcomes to HIV-negative children. Further studies are needed to identify gaps in PMTCT and PCP prophylaxis in HIV-infected and HEU children as well as the long-term outcomes of HIV-infected and HEU children admitted to PICU.

## Figures and Tables

**Table 1:** Comparison of HIV-negative and HIV-infected patients admitted to the PICU.

	HIV Negative	HIV Infected	*p*-Value
Total Patients	534	35	
Admission age (months) Median (IQR)	13 (51)	2 (1)	***p* < 0.001**
Weight z-score Median (IQR)	−1.45 (3.20)	−2.42 (2.33)	*p* = 0.063
Main Admission Diagnosis n (%):			***p* < 0.001**
Surgical	257/534 (48.1)	2/35 (5.7)	
A, P and D	85/534 (15.9)	0/35 (0)	
LRT	100/534 (18.7)	29/35 (82.9)	
Other medical	92/534 (17.2)	4/35 (11.4)	
Viral Infection n (%)	25/517 (4.8)	14/34 (41.2)	***p* < 0.001**
PCP/CMV n (%):			***p* < 0.001**
PCP only	2/532 (0.4)	13/35 (37.1)	
CMV only	1/532 (0.2)	2/35 (5.7)	
PCP + CMV	0/532 (0)	10/35 (28.6)	
TB n (%)	8/531 (1.5)	6/35 (17.1)	***p* < 0.001**
Ventilation mode n (%):			***p* < 0.001**
CPAP	15/516 (2.9)	0/33 (0)	
IPPV	426/516 (82.6)	23/33 (69.7)	
HFOV	23/516 (4.5)	10/33 (30.3)	
ICU duration (days) Median (IQR)	3 (5)	9 (10)	***p* < 0.001**
Ventilation length (days) Median (IQR)	2 (5)	7.5 (10)	***p* < 0.001**
Died in ICU n (%)	120/529 (22.7)	14/35 (40)	***p* = 0.02**

HEU = HIV exposed uninfected; IQR = interquartile range; A, D and P = accidents, drownings and poisonings; LRT = lower respiratory tract; PCP = pneumocystis pneumonia; CMV = cytomegalovirus; TB = tuberculosis; CPAP = continuous positive airway pressure; IPPV = intermittent positive pressure ventilation; HFOV = high-frequency oscillatory ventilation.

**Table 2:** Comparison of HIV-negative and HEU patients admitted to the PICU.

	HIV Negative	HEU	*p*-Value
Total Patients	534	109	
Admission age (months) Median (IQR)	13 (51)	3 (6)	***p* < 0.001**
Weight z-score Median (IQR)	−1.45 (3.20)	−2.71 (4.34)	***p* = 0.001**
Main Admission Diagnosis n (%):			***p* < 0.001**
Surgical	257/534 (48.1)	39/109 (35.8)	
A, P and D	85/534 (15.9)	2/109 (1.8)	
LRT	100/534 (18.7)	43/109 (39.4)	
Other medical	92/534 (17.2)	25/109 (22.9)	
Viral Infection n (%)	25/517 (4.8)	12/106 (11.3)	***p* < 0.001**
PCP/CMV n (%):			***p* < 0.001**
PCP only	2/532 (0.4)	5/109 (4.6)	
CMV only	1/532 (0.2)	2/109 (1.8)	
PCP + CMV	0/532 (0)	2/109 (1.8)	
TB n (%)	8/531 (1.5)	4/109 (3.7)	*p* = 0.129
Ventilation mode n (%):			*p* = 0.163
CPAP	15/516 (2.9)	4/105 (3.8)	
IPPV	426/516 (82.6)	83/105 (79)	
HFOV	23/516 (4.5)	10/105 (9.5)	
ICU duration (days) Median (IQR)	3 (5)	4 (8)	*p* = 0.163
Ventilation length (days) Median (IQR)	2 (5)	3.5 (7)	*p* = 0.443
Died in ICU n (%)	120/529 (22.7)	33/109 (30.3)	*p* = 0.091

HEU = HIV exposed uninfected; IQR = interquartile range; A, D and P = accidents, drownings and poisonings; LRT = lower respiratory tract; PCP = pneumocystis pneumonia; CMV = cytomegalovirus; TB = tuberculosis; CPAP = continuous positive airway pressure; IPPV = intermittent positive pressure ventilation; HFOV = high-frequency oscillatory ventilation.

**Table 3:** Comparison of HEU and HIV-infected patients admitted to the PICU.

	HEU	HIV Infected	*p*-Value
Total Patients	109	35	
Admission age (months) Median (IQR)	3 (6)	2 (1)	*p* = 1.00
Weight z-score Median (IQR)	−2.71 (4.34)	−2.42 (2.33)	*p* = 1.00
Main Admission Diagnosis n (%):			***p* < 0.001**
Surgical	39/109 (35.8)	2/35 (5.7)	
A, P and D	2/109 (1.8)	0/35 (0)	
LRT	43/109 (39.4)	29/35 (82.9)	
Other medical	25/109 (22.9)	4/35 (11.4)	
Viral Infection n (%)	12/106 (11.3)	14/34 (41.2)	***p* < 0.001**
PCP/CMV n (%):			***p* < 0.001**
PCP only	5/109 (4.6)	13/35 (37.1)	
CMV only	2/109 (1.8)	2/35 (5.7)	
PCP + CMV	2/109 (1.8)	10/35 (28.6)	
TB n (%)	4/109 (3.7)	6/35 (17.1)	***p* = 0.006**
Ventilation mode n (%):			***p* = 0.009**
CPAP	4/105 (3.8)	0/33 (0)	
IPPV	83/105 (79)	23/33 (69.7)	
HFOV	10/105 (9.5)	10/33 (30.3)	
ICU duration (days) Median (IQR)	4 (8)	9 (10)	***p* = 0.009**
Ventilation length (days) Median (IQR)	3.5 (7)	7.5 (10)	***p* = 0.001**
Died in ICU n (%)	33/109 (30.3)	14/35 (40)	*p* = 0.286

HEU = HIV exposed uninfected; IQR = interquartile range; A, D and P = accidents, drownings and poisonings; LRT = lower respiratory tract; PCP = pneumocystis pneumonia; CMV = cytomegalovirus; TB = tuberculosis; CPAP = continuous positive airway pressure; IPPV = intermittent positive pressure ventilation; HFOV = high-frequency oscillatory ventilation.

**Table 4:** Comparison by HIV status in children admitted to PICU with medical conditions.

	HIV Negative	HEU	HIV Infected	*p*-Value
Weight z-score Median (IQR)	−2.11 (3.13)	−2.37 (4.48)	−2.42 (2.33)	0.338
Age (months) Median (IQR)	5.5 (22)	3 (6)	2 (1)	**0.001**
Duration ventilation Median (IQR)	4 (6)	4 (7)	8 (10)	**0.001**
Duration ICU Median (IQR)	4 (7)	5 (9)	9 (10)	**0.001**
Died in ICU (%)	62/190 (32.6)	27/68 (39.7)	14/33 (42.4)	0.387

HEU = HIV exposed uninfected; IQR = interquartile range.

## Data Availability

The datasets collected and analysed during the study are available from the corresponding author on reasonable request.

## Abbreviations

A, D and P	Accidents, drownings and poisonings
ART	Antiretroviral Therapy
CMJAH	Charlotte Maxeke Johannesburg Academic Hospital
CMV	Cytomegalovirus
CPAP	Continuous Positive Airway Pressure
ELISA	Enzyme-Linked Immunosorbent Assay
HEU	HIV Exposed Uninfected
HFOV	High-Frequency Oscillatory Ventilation
HIV	Human Immunodeficiency Virus
ICU	Intensive Care Unit
IPPV	Intermittent Positive Pressure Ventilation
IQR	Interquartile Range
LDH	Lactate Dehydrogenase
LMICs	Low- and Middle-Income Countries
LRT	Lower Respiratory Tract
PCR	Polymerase Chain Reaction
PICU	Paediatric Intensive Care Unit
PIM3	Paediatric Index of Mortality 3
PCP	Pneumocystis Pneumonia
PMTCT	Prevention of Mother-to-Child Transmission
REDCap	Research Electronic Data Capture
SPSS	Statistical Programme for the Social Sciences
WHO	World Health Organization
